# Activation and expression of endogenous CREB‐regulated transcription coactivators (CRTC) 1, 2 and 3 in the rat adrenal gland

**DOI:** 10.1111/jne.12920

**Published:** 2020-12-14

**Authors:** Lorna I. F. Smith, Zidong Zhao, Jamie Walker, Stafford Lightman, Francesca Spiga

**Affiliations:** ^1^ Bristol Medical School: Translational Health Sciences University of Bristol Bristol UK; ^2^ College of Engineering, Mathematics and Physical Sciences University of Exeter Exeter UK; ^3^ EPSRC Centre for Predictive Modelling in Healthcare University of Exeter Exeter UK

**Keywords:** adrenal cortex, CREB, CRTC, StAR, steroidogenesis

## Abstract

The activation and nuclear translocation of cAMP‐response element binding protein (CREB)‐regulated transcription coactivator (CRTC)2 occurs in the rat adrenal gland, in response to adrenocorticotrophic hormone (ACTH) and stressors, and has been implicated in the transcriptional regulation of steroidogenic acute regulatory protein (StAR). We have recently demonstrated the activation of CRTC isoforms, CRTC1 and CRTC3, in adrenocortical cell lines. In the present study, we aimed to determine the activation and expression of the three CRTC isoforms in vivo in relation to *Star* transcription, under basal conditions and following a robust endotoxic stress challenge. Rat adrenal glands and blood plasma were collected following i.v. administration of either an ultradian‐sized pulse of ACTH or administration of lipopolysaccharide, as well as under unstressed conditions across the 24‐hour period. Plasma ACTH and corticosterone (CORT) were measured and the adrenal glands were processed for measurement of protein by western immunoblotting, RNA by a quantitative reverse transcriptase‐polymerase chain reaction and association of CRTC2 and CRTC3 with the *Star* promoter by chromatin immunoprecipitation. An increase in nuclear localisation of CRTC2 and CRTC3 followed increases in both ultradian and endotoxic stress‐induced plasma ACTH, and this was associated with increased CREB phosphorylation and corresponding increases in *Star* transcription. Both CRTC2 and CRTC3 were shown to associate with the *Star* promoter, with the dynamics of CRTC3 binding corresponding to that of nuclear changes in protein levels. CRTC isoforms show little variation in ultradian expression or variation across 24 hours, although evidence of long‐term down‐regulation following endotoxic stress was found. We conclude that co‐transcription factors CRTC2 and, more clearly, CRTC3 appear to act alongside phosphorylated CREB in the generation of ultradian pulses of *Star* transcription, essential for the maintenance of basal StAR expression. Similarly, our findings suggest CRTC2 and CRTC3 mediate *Star* transcriptional initiation following an endotoxic stressor; however, other transcription factors are likely to be responsible for the long‐term up‐regulation of adrenal *Star* transcription.

## Graphical abstract

The present study aimed to determine the in vivo dynamics of activation and expression of the three cAMP‐response element binding protein (CREB)‐regulated transcription coactivator (CRTC) isoforms in relation to adrenocorticotrophic hormone (ACTH)‐induced steroidogenic acute regulatory (StAR) transcription. Nuclear localisation and association of CRTC2 and CRTC3 at the Star promoter followed increases in plasma ACTH induced by ultradian and endotoxic stress activity. Corresponding with increased CREB phosphorylation and increases in Star transcription, we conclude that CRTC2 and CRTC3 appear to play a role as co‐transcription factors alongside phosphorylated CREB in the initiation of Star transcription.

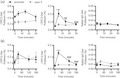

## 1 INTRODUCTION

The release of glucocorticoid hormones (corticosterone in rodents and as cortisol in humans, here referred as CORT) is tightly regulated by the hypothalamic‐pituitary‐adrenal (HPA) axis, enabling the body to adapt in response to stress alongside the autonomic nervous system.^1^ Glucocorticoids are secreted from the adrenal gland, following stimulation by adrenocorticotrophic hormone (ACTH) from the anterior pituitary, which in turn is stimulated by corticotrophin‐releasing hormone (CRH) secreted by paraventricular neurones of the hypothalamus.

Within steroidogenic zona fasciculata cells of the adrenal cortex, CORT is synthesised de novo upon ACTH activation of steroidogenic enzymes. The activity and expression of these enzymes, particularly that of rate‐limiting steroidogenic acute regulatory (StAR) protein, is tightly regulated by ACTH, predominantly through phosphorylation by the cAMP/PKA pathway.^2^, ^3^, ^4^, ^5^ Turnover of StAR protein is rapid, with the active form of StAR having a half‐life of approximately 5‐15 minutes.^3^, ^6^ In addition to rapid activation of StAR and other steroidogenic enzymes through phosphorylation, ACTH also stimulates the transcription of *Star* and the steroidogenic enzymes to replenish and maintain expression.^7^, ^8^, ^9^

A key transcription factor involved in the regulation of *Star* transcription is cAMP response element binding protein (CREB), directly regulated by ACTH through phosphorylation.^10^, ^11^ Additionally, ACTH can indirectly activate CREB through post‐transcriptional modification of co‐transcription factors, including the well‐characterised CREB binding protein/p300 (CBP/p300) and CREB‐regulated transcription coactivator (CRTC, previously known as TORC). CRTC is a co‐transcription factor that enhances binding of CREB to the gene promoter through its binding to the CREB bZIP domain, working independently of CBP.^12^ CRTC is sequestered in the cytoplasm until activated by dephosphorylation, following increased intracellular cAMP levels.^13^, ^14^ This allows CRTC to translocate into the nucleus and bind CREB at the transcription site.^15^ Three isoforms (CRTC1, 2 and 3) have been identified, with CRTC2 and CRTC3 shown to be the most highly expressed in the adrenal gland.^12^, ^16^ CRTC2 has long been implicated in the regulation of *Star* transcription in vitro^13^, ^14^ and in vivo, where nuclear levels of phosphorylated CREB (pCREB) and CRTC2 in the rat adrenal gland have been shown to increase in response to both high and low dose ACTH and restraint stress.^17^, ^18^ More recently, using murine adrenocortical cell lines, we have demonstrated that, in addition to CRTC2, ACTH also stimulates rapid nuclear translocation of CRTC1 and CRTC3, and that both CRTC2 and CRTC3 bind at the *Star* promoter in response to ACTH, suggesting a role for these isoforms in mediating the initiation of *Star* transcription.^16^

Under basal (unstressed) conditions, both ACTH and CORT levels fluctuate in both an ultradian and a circadian manner^19^ and we have shown that circadian and ultradian dynamics are evident within the steroidogenic pathway (both at the level of protein activation and gene transcription) and reflect ACTH and CORT profiles.^20^, ^21^ Furthermore, we have shown that the adrenal gland steroidogenic pathway is also activated in response to endotoxic stress.^21^

The present study aimed to examine whether the activity and expression of the endogenous CRTC isoforms in vivo in the rat adrenal gland exhibit a similar pattern of activity and expression as observed for other key regulators of CORT synthesis. Specifically, given the differential activation across CRTC isoforms observed in vitro, we aimed to determine the extent to which endogenously expressed CRTC1, CRTC2 and CRTC3 may be activated by translocation and associated with the *Star* promoter during the initiation and sustained transcription of *Star* in response to an ACTH ultradian pulse and following acute endotoxic stress. Furthermore, because several key steroidogenic regulators exhibit an ultradian and circadian pattern of expression in basal condition, as well as in response to stress, we investigated whether CRTC 1‐3 also exhibit strong endogenous regulation of transcription and expression.

## 2 MATERIALS AND METHODS

### 2.1 Animals

All experiments were conducted in adult male Sprague‐Dawley rats (7‐8 weeks old and weighing 200‐250 g; MGI catalogue no. 5651135; RRID: MGI: 5651135; Harlan Laboratories, Inc, Blackthorn, UK). Animals were given a 1‐week period of acclimatisation prior to surgery or the commencement of experimental procedures. All animals were maintained under a 14:10 hour light/dark photocycle (lights on at 5.00 am) at 21 ± 1°C with access to food and water available ad lib. Rats were housed four to a cage but were singly housed following cannulation surgery. All animal procedures were approved by Animal Welfare Ethical Review board of University of Bristol and conducted in accordance with Home Office guidelines and the UK Animals (Scientific Procedures) Act, 1986 (Project Licence number: 30/3043).

### 2.2 Experimental procedures and tissue collection

In the ultradian ACTH stimulation and lipoploysaccharide (LPS) injection experiments, rats were surgically implanted with an indwelling cannula in the jugular vein to allow for i.v. injections, under anaesthetic with a combination of Hypnorm (0.32 mg kg^‐1^ fentanyl citrate and 10 mg kg^‐1^ fluanisone, i.m.; Janssen Pharmaceuticals, Oxford, UK) and diazepam (2.6 mg kg^‐1^ i.p.; Phoenix Pharmaceuticals, Gloucester, UK), as described previously.^22^ Following 5‐7 days recovery after surgery, experiments were commenced at 9.00 am. Rats were administered with either an ultradian‐sized ACTH pulse (10 ng in a 100‐µL volume of 0.9% saline solution; Alliance Pharmaceuticals, Ltd, Chippenham, UK; n = 5‐7 independent rats per group) or LPS (*Escherichia coli*; 055: B5; 25 µg in a 100‐µL volume of 0.9% saline solution; Sigma‐Aldrich, St Louis, MO, USA; n = 4‐6 independent rats per group) via their indwelling catheter. Rats were overdosed with 0.2 mL of Euthatal (200 mg mL^‐1^ sodium pentobarbital; Merial, Harlow, UK) via the indwelling cannula at the time points indicated.

For investigation of am‐pm variation, rats were maintained under a normal light/dark schedule as described above. Rats were killed using isoflurane every 4 hours at 1.00 am, 5.00 am, 9.00 am, 1.00 pm, 5.00 pm and 9.00 pm (n = 5 or 6 per time point).

Immediately after death, as indicated above, trunk blood was collected following decapitation. Blood was then processed for measurement by radioimmunoassay of plasma ACTH (DiaSorin, Stillwater, MN, USA) and corticosterone, as described previously.^22^ Adrenal glands were collected and the outer capsule (containing the zona glomerulosa) was removed to obtain the inner zones (comprising the zona fasciculata and zona reticularis of the cortex and the medulla). These were then frozen immediately for protein extraction or RNA isolation or processed immediately for a chromatin immunoprecipitation (ChIP) assay.

### 2.3 RNA isolation and quantitative reverse transcriptase‐polymerase chain reaction (qRT‐PCR)

RNA was extracted using TRIzol reagent and then purified using RNeasy mini kit reagents and column deoxyribonuclease digestion (Qiagen, Manchester, UK) to remove genomic DNA contamination. RNA was tested using a NanoDrop spectrophotometer (Thermo Fisher Scientific, Waltham, MA, USA) to check quantity via measurement of optical density at 260 nm and quality by via measurement of optical density at 260 nm (*A*_260_/*A*_280_ range 1.8‐2.1) and 230 nm (*A*_260_/*A*_230_ range > 1.5). cDNA was reverse transcribed from 1 µg of total RNA using an AMV first‐strand synthesis system kit (Invitrogen, Carlsbad, CA, USA). qPCR primers were designed for both primary transcripts, spanning an intronic‐exonic region to detect heteronuclear RNA (hnRNA) and mature mRNA, as shown in Table 1. PCR reactions were performed using Fast SYBR Green master mix (Applied Biosystems, Foster City, CA, USA) at 166 nmol L^‐1^ for each primer and 2 μL of cDNA, with a final volume of 15 μL, in a StepOne Plus RT‐PCR machine (Applied Biosystems). Briefly, samples underwent denaturation at 50°C for 2 minutes, 95°C for 20 seconds, followed by 40 cycles at 95°C for 3 seconds and 60°C for 30 seconds. Levels were calculated using relative quantification by standard curve, normalised to glyceraldehyde 3‐phosphate dehydrogenase (*Gapdh*) mRNA, as determined in separate qRT‐PCR reactions. *Gapdh* mRNA stability was confirmed by statistical analysis, as described below. Absence of detection when omitting the reverse transcription enzyme Superscript III (Invitrogen) indicated a lack of genomic DNA contamination.

**Table 1:** Rat primer sequences

RNA	Target	Primer	Sequence	Product (bp)
hnRNA	Star	Forward	GCAGCAGCAACTGCAGCACTAC	114
		Reverse	GTGCCCCCGGAGACTCACCT	
	Crtc1	Forward	TATCCACTGATCTCCCCAGTCTC	194
		Reverse	AGCCTCCTGTGTTGTGGGTAG	
	Crtc2	Forward	CCCTTGCCTTTCTCGTCCATT	240
		Reverse	CCCAGCAGTGGGGTATTCA	
	Crtc3	Forward	ACACAAAGCACCAATATGCAGT	160
		Reverse	CGGGTCCCACGGACATTATC	
mRNA	Star	Forward	CTGGCAGGCATGGCCACACA	161
		Reverse	GGCAGCCACCCCTTGAGGTC	
	Crtc1	Forward	CACCAGAGCACAATGACACC	161
		Reverse	GCCTTCTTTGAGTCCCATGA	
	Crtc2	Forward	CCCACCCCAAAGTCTCTACA	168
		Reverse	CCCCAGGCTGAAGTCATTTA	
	Crtc3	Forward	AAGCCAGGTACCCTCCAACT	162
		Reverse	GCACATACAGGAAAGCAGCA	
	Gapdh	Forward	TGCACCACCAACTGCTTA	231
		Reverse	GGATGCAGGGATGATGTTC	

### 2.4 Protein extraction and western immunoblotting

Whole cell lysate extraction was performed as described previously.^20^ For extraction of nuclear and cytosolic protein, a dounce homogeniser (Kontes; Kimble Chase, Vineland, NJ, USA) was used to homogenise the adrenal glands on ice with a loose pestle for 15‐20 strokes until homogenously mixed. Nuclear and cytosolic proteins were then extracted from the homogenised adrenal glands using the NE‐PER Nuclear and Cytoplasmic Extraction Reagent kit (Pierce, Rockford, IL, USA) with Inhibitors Cocktail and 0.5 mol L^‐1^ ethylenediaminetetraacetic acid (Halt; Thermo Fisher Scientific) in accordance with the manufacturer’s instructions. Nuclear and cytosolic extract from each sample (13 µg) were separated in Tris‐Glycine gels, transferred to polyvinylidene fluoride membranes and blocked for 1 hour in Tris‐buffered saline‐Tween 20 with 5% milk. Immunoblots were then incubated at 4°C, as indicated in Table 2. Membranes were washed and incubated with a horseradish peroxidase‐conjugated donkey anti‐rabbit immunoglobulin (Ig)G (dilution 1:10 000; catalogue no. A120‐108P; RRID: AB_10892625; Bethyl, Montogomery, TX, USA) or horseradish peroxidase‐conjugated donkey anti‐goat IgG (dilution 1:5000; catalogue no. A50‐101P; RRID: AB_66755; Bethyl). Detection of immunoreactive bands was performed using Luminata Forte Western HRP substrate (Millipore, Burlington, MA, USA) followed by imaging using G:BOX Chemi XX6 system (Syngene, Bangalore, India). Band intensity was semi‐quantified using ImageJ (NIH, Bethesda, MD, USA). The results are expressed as fold‐change over the control values after correction for protein loading using α‐tubulin and GAPDH for whole cell, GAPDH and vinculin for cytoplasm, or histone deacetylase 1 and vinculin for nuclear extracts, as indicated. Normalisation of protein expression was achieved by dividing the integrated density of the protein band by the average integrated density of the protein bands of the two housekeeping genes used for the same sample. Housekeeping gene stability was confirmed by statistical analysis as described below. Robustness of normalisation was confirmed by additional analysis of nuclear and whole cell protein levels normalised to each housekeeping gene individually (see Supporting information, Tables S1‐S3).

**Table 2:** Western immunoblotting antibodies

Target	Size (kDa)	Dilution	Antibody Species	Company
CRTC1	78	1:500 in 5% BSA overnight	Rabbit	Cell Signaling Technology, catalogue no. 2501, RRID: AB_659914
CRTC2	79‐83	1:5000 in TBST overnight	Rabbit	Millipore, catalogue no. ST1099, RRID: AB_2276561
CRTC3	76	1:1000 in TBST overnight	Rabbit	Cell Signaling Technology, catalogue no. 2720, RRID: AB_2083845
pCREB	43	1:1000 in TBST overnight	Rabbit	Cell Signaling Technology, catalogue no. 9198, RRID: AB_2561044
CREB	43	1:500 in 5% BSA overnight	Rabbit	Cell Signaling Technology, catalogue no. 9197, RRID: AB_331277
GAPDH	36	1:7500 in TBST overnight	Rabbit	Cell Signaling Technology, catalogue no. 5014, RRID: AB_10693448
HDAC1	60	1:1000 in TBST overnight	Goat	Santa Cruz Biotechnology, catalogue no. sc‐6298, RRID: AB_2279712
α‐Tubulin	52	1:1000 in TBST overnight	Rabbit	Cell Signaling Technology, catalogue no. 2144, RRID: AB_2210548
Vinculin	117	1:5000 in TBST for 1 h	Goat	Santa Cruz Biotechnology, catalogue no. sc‐7649, RRID: AB_2288413

Note

BSA, bovine serum albumin; CREB, cAMP‐response element binding protein; CRTC, CREB‐regulated transcription coactivator; HDAC1, histone deacetylase 1; TBST, Tris‐buffered saline‐Tween 20.

### 2.5 ChIP assay and qPCR analysis

Immediately following collection adrenal glands (n = 3‐4 independent rats per group) were fixed and chromatin was extracted as described previously.^23^ Eighty‐five micrograms of sheared chromatin were immunoprecipitated as described previously,^16^ with either anti‐TORC2 antibody (1 µg mL^‐1^; catalogue no. ab109081; RRID: AB_10859591; Abcam, Cambridge, MA, USA), anti‐CRTC3 antibody (2 µg mL^‐1^; catalogue no. ab91654; RRID: AB_2049542; Abcam) or ChIP‐grade non‐specific rabbit IgG (4 μg mL^‐1^; catalogue no. 2729; RRID: AB_1031062: Cell Signaling Technology, Beverly, MA, USA). Samples were stored at −80°C prior to DNA quantification by qRT‐PCR, as described above. Primers spanning the three putative CRE sites at −210 bp to −37 bp of the *Star* promoter (forward 5'‐AAGTTATGCCCTTTGCCCCA‐3', reverse 5'‐CGGAAGGCTGTGCATCATCA‐3', Invitrogen) or exon 5 of the *Star* gene (forward 5'‐CGCTGTACCAAGCGTAGAGG‐3', reverse 5'‐CAGGCATCTCCCCAAAGTGT‐3', 70 bp product), used as a negative control, were designed using NCBI primer‐blast (https://www.ncbi.nlm.nih.gov/tools/primer-blast) as determined via NCBI GenBank (https://www.ncbi.nlm.nih.gov/genbank). Promoter pulldown was quantified using rat genomic DNA, normalised to the total levels in the chromatin input (promoter content in chromatin not subjected to immunoprecipitation).

### 2.6 Statistical analysis

For these experiments, no animals were excluded from the study. Rats were allocated to treatment/timepoint groups using randomisation within blocks, where each block represents a different procedural day. Researchers were blind to treatment/timepoint allocation when performing a radioimmunoassay for plasma ACTH and corticosterone measurement and for RNA quantification by qRT‐PCR. Western immunoblot gels were not blinded, aiming to ensure that, where possible, each time point was represented on each gel; however, quantification of proteins bands was performed using ImageJ; thus, the risk of bias was minimised. All data are expressed as the mean ± SEM of values obtained from a minimum of three independent experiments. Normal Q‐Q plots were used to ensure that data appeared normally distributed, whilst homogeneity of variance was verified using the Bartlett's test. Where data showed equal variance, they were analysed using one‐way analysis of variance (ANOVA) or two‐way ANOVA, as indicated. Where one‐way ANOVA or two‐way ANOVA was significant, this was followed by Tukey’s post‐hoc analysis to compare all means with every other mean. Where Bartlett's test showed significant variance between data groups, Welch’s ANOVA was used, as indicated. Where Welch’s ANOVA was significant, this was followed by Games‐Howell post‐hoc analysis to compare all means with every other mean. The Games‐Howell test has a similar formulation to Tukey's test, although it does not assume equal variances and sample sizes. *P* < 0.05 was considered statistically significant, with a trend defined as *P* < 0.1. prism, version 8 (GraphPad Software Inc., San Diego, CA, USA) was used for the statistical analysis. For each am‐pm data set, a sine function (with a fixed 24 hours period) was numerically fitted to the experimental data in the least‐squares sense using matlab (MathWorks, Natick, MA, USA). The acrophase was then taken as the clock time corresponding to the peak value in the fitted curve.^20^

## 3 RESULTS

### 3.1 CRTC activation in response to an ultradian pulse of ACTH

**Figure 1:**
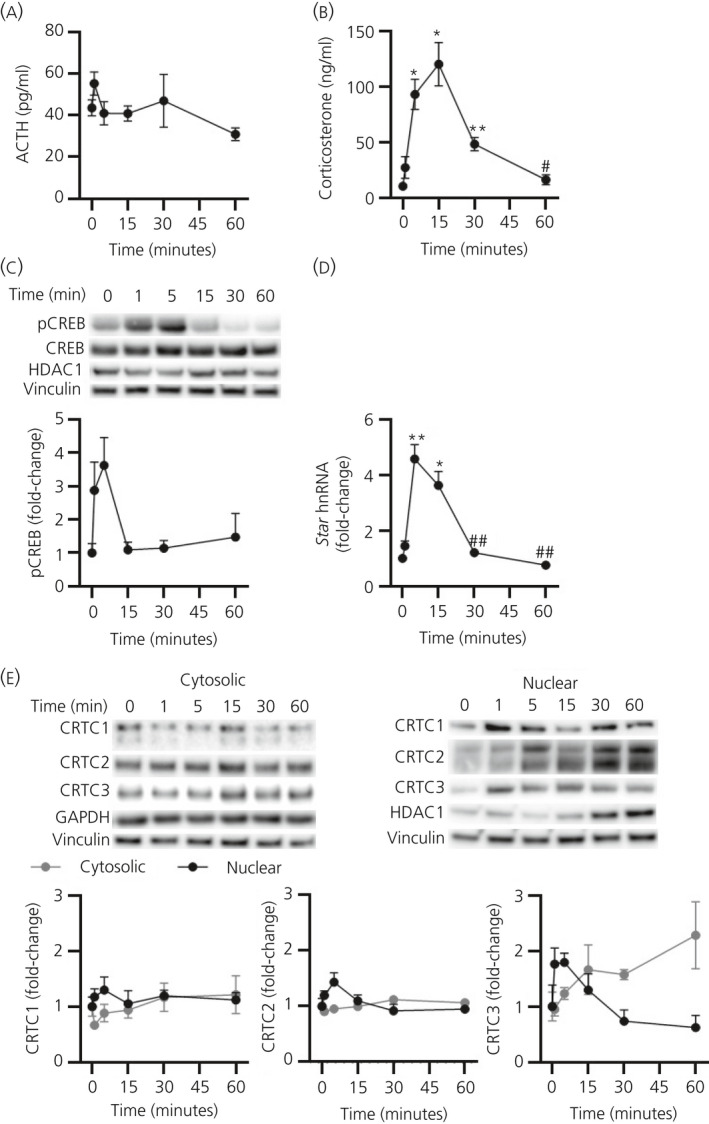
Effect of an ultradian pulse of adrenocorticotrophic hormone (ACTH) on cAMP‐response element binding protein (CREB)‐regulated transcription coactivator (CRTC)1‐3 activation. (A) Plasma ACTH (pg mL^‐1^), (B) plasma corticosterone (ng mL^‐1^), (C) a representative immunoblot of nuclear levels of phosphorylated CREB (pCREB) and total CREB and semi‐quantification of nuclear pCREB, normalised to histone deacetylase 1 (HDAC1) (*P* = 0.851 by one‐way analysis of variance [ANOVA]) and vinculin (*P* = 0.999 by one‐way ANOVA), (D) *Star* heteronuclear RNA (hnRNA), normalised to glyceraldehyde 3‐phosphate dehydrogenase (*Gapdh*) mRNA (*P* = 0.177 by one‐way ANOVA) and (E) a representative western immunoblot of cytosolic and nuclear CRTC1, CRTC2 and CRTC3 protein levels and semi‐quantification of CRTC1, CRTC2 and CRTC3 from cytosolic protein, normalised to GAPDH (*P* = 0.998 by one‐way ANOVA) and vinculin (*P* = 0.292 by one‐way ANOVA) and nuclear protein, normalised to HDAC1 and vinculin (as above), following 10 ng of i.v. ACTH administration. Data are represented as the mean ± SEM (n = 5‐7 independent rats per group); data in (C) to (E) are expressed as fold induction of time 0. ACTH, corticosterone, pCREB, *Star* hnRNA and cytosolic CRTC1 and CRTC3 data were analysed using Welch’s ANOVA followed by Games‐Howell post‐hoc test; CRTC2 and nuclear CRTC1 and CRTC3 data were analysed using one‐way ANOVA followed by Tukey’s post‐hoc test. *P ≤ 0.05, **P ≤ 0.01 vs 0 min. #*P* ≤ 0.05, ##*P* ≤ 0.01 vs maximum levels

An ultradian pulse of ACTH was administered in rats (10 ng i.v.) to induce an adrenal response similar to that observed in basal (unstressed) conditions. As recently reported,^21^ a pulse of ACTH (*F*_5,14.14_ = 2.909; *P* = 0.052 overall by Welch’s ANOVA) (Figure 1A) induced a significant increase in plasma CORT (*F*_5,13.51_ = 14.59; *P* < 0.0001 overall by Welch’s ANOVA) (Figure 1B) that was paralleled by variable increases in pCREB (*F*_5,13.92_ = 2.216; *P* = 0.111 overall by Welch’s ANOVA) (Figure 1C) and *Star* hnRNA (*F*_5,12.82_ = 18.01; *P* < 0.0001 overall by Welch’s ANOVA) (Figure 1D). Similar experiments performed previously in our laboratory have established that administration of saline via the indwelling catheter elicits no such response in plasma ACTH and CORT levels.^24^ Ultradian activity of CRTC isoforms was investigated by isolating and quantifying the adrenal gland nuclear cell fraction to measure activation by nuclear translocation (Figure 1E). ACTH had no significant effect on cytosolic levels of CRTC1 or CRTC2, although it did affect CRTC3 levels, which were maximal by 60 minutes following ACTH administration (CRTC1 *F*_5,13.71_ = 1.638 *P* = 0.216 overall by Welch’s ANOVA; CRTC2 *F*_5,31_ = 1.841 *P* = 0.134 overall by one‐way ANOVA; CRTC3 *F*_5,14.05_ = 3.746 *P* = 0.023 overall by Welch’s ANOVA) (Figure 1E). Although there was no effect of ACTH on nuclear levels of CRTC1 (*F*_5,31_ = 0.383; *P* = 0.857 overall by one‐way ANOVA), we found a significant effect on nuclear levels of both CRTC2 (*F*_5,31_ = 2.532; *P* = 0.049 overall by one‐way ANOVA) and CRTC3 (*F*_5,31_ = 3.232; *P* = 0.018 overall by one‐way ANOVA). Nuclear levels of CRTC2 and CRTC3 were maximal at 5 minutes after the ACTH injection, although this increase was not significant by pairwise analysis (CRTC2 *P* = 0.126 0 minute vs 5 minutes by Tukey’s post‐hoc test, CRTC3 *P* = 0.326 0 minute vs 5 minutes by Tukey’s post‐hoc test); however, the subsequent decrease in nuclear CRTC2 and CRTC3 levels showed a trend for significance (CRTC2 *P* = 0.054 5 minutes vs 30 minutes by Tukey’s post‐hoc test; CRTC3 *P* = 0.061 5 minutes vs 60 minutes by Tukey’s post‐hoc test).

### 3.2 CRTC activation in response to an endotoxic stressor

**Figure 2:**
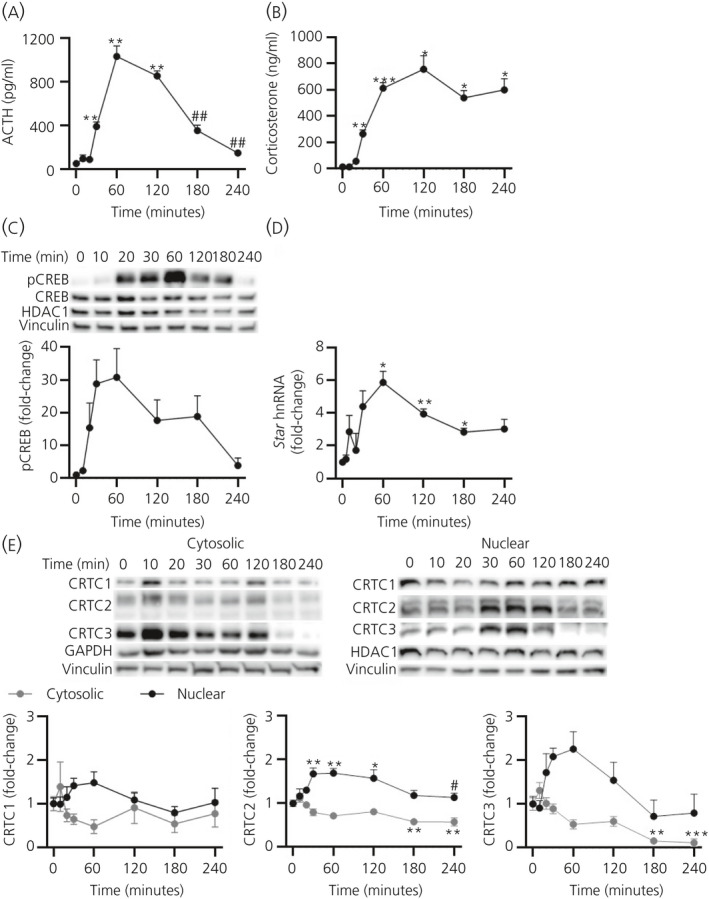
Effect of an endotoxic stressor on cAMP‐response element binding protein (CREB)‐regulated transcription coactivator (CRTC)1‐3 activation. (A) Plasma adrenocorticotrophic hormone (ACTH) (pg mL^‐1^), (B) plasma corticosterone (ng mL^‐1^), (C) a representative immunoblot of nuclear levels of phosphorylated CREB (pCREB) and total CREB and semi‐quantification of nuclear pCREB, normalised to histone deacetylase 1 (HDAC1) (*P* = 0.998 by one‐way one‐way analysis of variance [ANOVA]) and vinculin (*P* = 0.913 by one‐way ANOVA), (D) *Star* heteronuclear RNA (hnRNA), normalised to glyceraldehyde 3‐phosphate dehydrogenase (*Gapdh*) mRNA (*P* = 0.666 by one‐way ANOVA) and (E) a representative western immunoblot of cytosolic and nuclear CRTC1, CRTC2 and CRTC3 protein levels and semi‐quantification of CRTC1, CRTC2 and CRTC3 from cytosolic protein, normalised to GAPDH (*P* = 0.671 by one‐way ANOVA) and vinculin (*P* = 0.997 by one‐way ANOVA), and nuclear protein, normalised to HDAC1 and vinculin (as above), following 25 μg of i.v. lipopolysaccahride administration. Data are represented as the mean ± SEM (n = 4‐6 independent rats per group); data in (C) to (E) are expressed as fold induction of time 0. ACTH, corticosterone, pCREB and *Star* hnRNA data were analysed using Welch’s ANOVA followed by Games‐Howell post‐hoc test; CRTC1, CRTC2 and CRTC3 data were analysed using one‐way ANOVA followed by Tukey’s post‐hoc test. *P ≤ 0.05, **P ≤ 0.01, ***P ≤ 0.001 vs 0 min. #*P* ≤ 0.05, ##*P* ≤ 0.01 vs maximum levels

To activate the adrenal gland response to endotoxic stress, rats were injected i.v. with LPS (Figure 2). As previously reported, there was a significant increase in plasma ACTH (*F*_7,11.79_ = 50.25; *P* < 0.0001 overall by Welch’s ANOVA) (Figure 2A), CORT (*F*_7,11.65_ = 49.71; *P* < 0.0001 overall by Welch’s ANOVA) (Figure 2B) and in adrenal gland levels of pCREB (*F*_7,11.53_ = 5.112; *P* = 0.008 overall by Welch’s ANOVA) (Figure 2C). This was paralleled by an increase in adrenal gland *Star* hnRNA levels (*F*_8,13.47_ = 15.55; *P* < 0001 overall by Welch’s ANOVA) (Figure 2D). Similar to the ultradian ACTH pulse experiment, dynamic changes of adrenal cytosolic and nuclear levels of CRTC1, CRTC2 and CRTC3 were measured following LPS (Figure 2E). Cytosolic levels of CRTC1 were not significantly altered (*F*_7,32_ = 1.296; *P* = 0.283 overall by one‐way ANOVA), although levels of CRTC2 (*F*_7,32_ = 6.845; *P* < 0.0001 overall by one‐way ANOVA) and CRTC3 (*F*_7,32_ = 10.10; *P* < 0.0001 overall by one‐way ANOVA) were significantly decreased by 180‐240 minutes (CRTC2 *P* = 0.006 0 minute vs 180 minutes by Tukey’s post‐hoc test; CRTC3 *P* = 0.001 0 minute vs 180 minutes by Tukey’s post‐hoc test). Changes in nuclear levels of CRTC1 were not detected (*F*_7,32_ = 1.193; *P* = 0.335 overall by one‐way ANOVA), whereas there was a significant increase in nuclear localisation of CRTC2 (*F*_7,32_ = 5.636; *P* = 0.0003 overall by one‐way ANOVA) and CRTC3 (*F*_7,32_ = 3.210; *P* = 0.011 overall by one‐way ANOVA). Nuclear levels of CRTC2 were maximal by 30 minutes (*P* = 0.002 0 minute vs 30 minutes by Tukey’s post‐hoc test) and CRTC3 by 60 minutes, although this increase was not significant (*P* = 0.115 0 minute vs 60 minutes by Tukey’s post‐hoc test), with levels similar to basal by 180 minutes.

### 3.3 Interaction of CRTC2 and CRTC3 at the *Star* promoter

**Figure 3:**
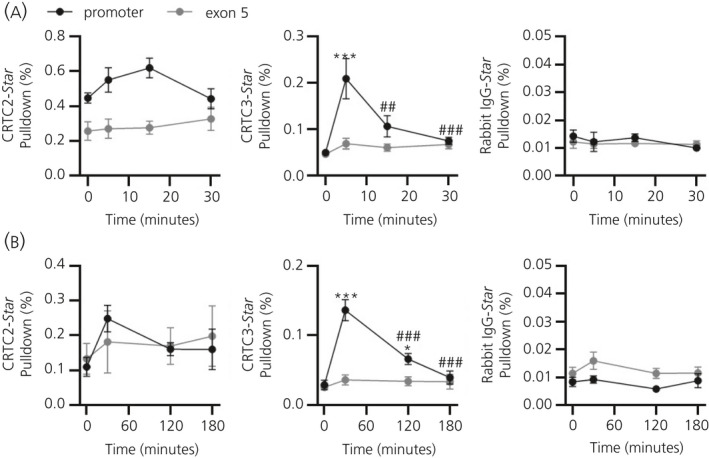
Association of cAMP‐response element binding protein (CREB)‐regulated transcription coactivator (CRTC)2 and CRTC3 at the *Star* promoter. Binding of CRTC2, CRTC3 and non‐specific rabbit immunoglobulin (Ig)G at the *Star* promoter and at *Star* exon 5 of the *Star* gene in the rat adrenal following (A) injection with 10 ng of adrenocorticotrophic hormone (ACTH), mimicking an ultradian ACTH pulse (n = 3‐4 independent rats per group) or (B) following endotoxic stress by injection with 25 μg of lipopolysaccahride (n = 4‐6 independent rats per group). Binding of these proteins was measured by a chromatin immunoprecipitation assay and quantified as the percentage pulldown of chromatin input. Data are represented as the mean ± SEM and analysed by two‐way one‐way analysis of variance (ANOVA) followed by Tukey’s post‐hoc test. *P ≤ 0.05, ***P ≤ 0.001 vs 0 min. ##*P* ≤ 0.01, ###*P* ≤ 0.001 vs maximum levels

To determine whether significant increases in adrenal nuclear CRTC2 and CRTC3 in response to ACTH were associated with increased binding of these isoforms at the *Star* promoter, ChIP assays were performed following injection of 10 ng of ACTH (Figure 3A) and 25 µg LPS (Figure 3B). Following an ultradian‐sized ACTH pulse (Figure 3A), CRTC2 binding at the *Star* promoter significantly increased compared to non‐specific binding at *Star* exon 5 (*F*_1,22_ = 33.45; gene region: *P* < 0.0001 by two‐way ANOVA); however, there was no significant change in levels of CRTC2 binding over time (time: *F*_3,22_ = 0.959 *P* = 0.430, interaction: *F*_3,22_ = 1.496; *P* = 0.243). For CRTC3, binding at the *Star* promoter was significantly higher than at exon 5 (*F*_1,22_ = 13.08; gene region: *P* = 0.002 by two‐way ANOVA) and this binding increased significantly following ACTH administration (*F*_3,22_ = 8.702; time: *P* = 0.0005), differing from that at exon 5 (interaction: *F*_3,22_ = 5.879; interaction: *P* = 0.004). CRTC3 pulldown of the *Star* promoter was maximal at 5 minutes, falling by 15 minutes. Comparatively, non‐specific pulldown of *Star* promoter was not significantly different from that of exon 5 by non‐specific rabbit IgG (*F*_1,22_ = 0.385; gene region: *P* = 0.541 by two‐way ANOVA), with no effect of time (*F*_3,22_ = 0.603; *P* = 0.620) or interaction (*F*_3,22_ = 0.283; *P* = 0.837). Following an endotoxic stressor (Figure 3B), CRTC2 did not appear to have increased pulldown of *Star* promoter compared to exon 5 (*F*_1,26_ = 0.0006; gene region: *P* = 0.981 by two‐way ANOVA) and there was no significant overall effect of time (*F*_3,26_ = 1.032; *P* = 0.395) or interaction (*F*_3,26_ = 0.369; *P* = 0.775). Within measurement of binding at the *Star* promoter only, however, there was a trend for an effect of time on increased CRTC2 binding (*F*_3,14_ = 2.589; *P* = 0.094 by one‐way ANOVA), with maximal pulldown at 30 minutes (*P* = 0.067 0 minute vs 30 minutes by Tukey’s post‐hoc test). CRTC3 binding following LPS administration was significantly increased at the *Star* promoter compared to exon 5 (*F*_1,30_ = 31.94; gene region: *P* < 0.0001 by two‐way ANOVA), with an overall significant effect of time (*F*_3,30_ = 19.20; *P* < 0.0001) and interaction (*F*_3,30_ = 14.19; *P* < 0.0001). Peak binding of CRTC3 to the *Star* promoter was seen at 30 minutes (*P* < 0.0001 0 minute vs 30 minutes by Tukey’s post‐hoc test), remaining partially elevated at 120 minutes (*P* = 0.025 0 minute vs 120 minutes by Tukey’s post‐hoc test). Again, non‐specific pulldown by rabbit IgG saw no significant effect of time (*F*_3,30_ = 1.252; *P* = 0.31 by two‐way ANOVA) or interaction (*F*_3,30_ = 0.426; *P* = 0.736), although pulldown of *Star* exon 5 was significantly elevated compared to that of the *Star* promoter (*F*_1,30_ = 9.136; gene region: *P* = 0.005).

### 3.4 Expression and transcriptional activation of CRTC1‐3

**Figure 4:**
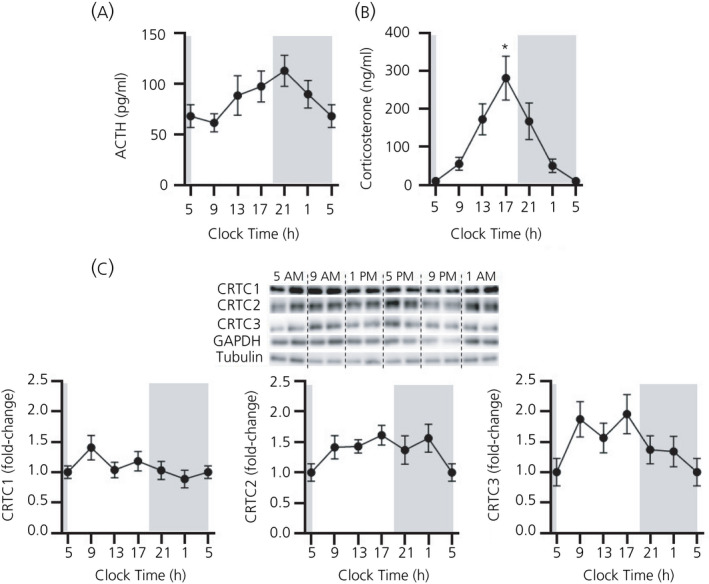
Variation in cAMP‐response element binding protein (CREB)‐regulated transcription coactivator (CRTC)1‐3 expression across 24 hours. (A) Plasma adrenocorticotrophic hormone (ACTH) (pg mL^‐1^), (B) plasma corticosterone (ng mL^‐1^) and (C) a representative western immunoblot of whole cell CRTC1, CRTC2 and CRTC3 protein levels and semi‐quantification of CRTC1, CRTC2 and CRTC3, normalised to α‐tubulin (*P* = 0.782 by one‐way one‐way analysis of variance [ANOVA]) and glyceraldehyde 3‐phosphate dehydrogenase (GAPDH) (*P* = 0.142 by one‐way ANOVA). For visualisation purposes, the value at 5.00 am has been plotted twice. The grey bar indicates the period of lights off (7.00 pm to 5.00 am). Data are represented as the mean ± SEM (n = 5 or 6 per time point); data in (C) are expressed as fold induction of levels at 5 am. ACTH and CRTC1, CRTC2 and CRTC3 protein data were analysed using one‐way ANOVA followed by Tukey’s post‐hoc test. Corticosterone data were analysed using Welch’s ANOVA followed by Games‐Howell post‐hoc test. *P ≤ 0.05 vs 5 am

In addition to activating steroidogenic transcriptional regulators, ACTH also regulates the transcription of many transcription factors involved in steroidogenesis, including SF‐1, Nur77 and DAX‐1. CRTC1, CRTC2 and CRTC3 protein levels were measured across the 24‐hour period (Figure 4C) in relation to plasma levels of ACTH (*F*_5,28_ = 1.693; *P* = 0.169 overall by one‐way ANOVA) (Figure 4A) and CORT (*F*_5,11.64_ = 9.076; *P* = 0.0009 overall by Welch’s ANOVA; acrophase: 16:54) (Figure 4B). No significant variation was detected for CRTC1, CRTC2 or CRTC3 protein expression (CRTC1: *F*_5,28_ = 1.364 *P* = 0.268 overall by one‐way ANOVA; CRTC2: *F*_5,28_ = 1.264; *P* = 0.307 overall by one‐way ANOVA; CRTC3: *F*_5,28_ = 1.739; *P* = 0.158) (Figure 4C).

**Figure 5:**
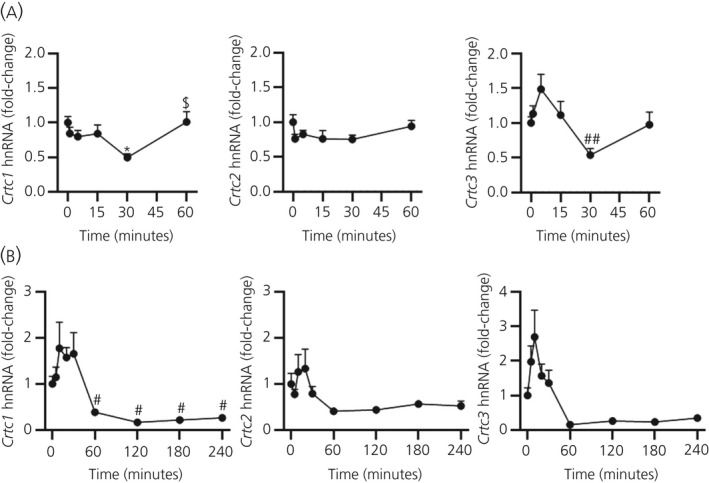
Effect of hypothalamic‐pituitary‐adrenal activation on cAMP‐response element binding protein (CREB)‐regulated transcription coactivator (CRTC)1‐3 transcription. Levels of rat adrenal Crtc1, Crtc2 and Crtc3 heteronuclear RNA (hnRNA) were measured by a quantitative reverse transcriptase‐polymerase chain reaction, normalised to *Gapdh* mRNA (A) following injection with 10 ng of adrenocorticotrophic hormone or (B) following injection with 25 μg of lipopolysaccahride. Data are represented as the mean ± SEM (n = 4‐7 independent rats per group), expressed as fold induction of time 0. Data in (A) were analysed using one‐way one‐way analysis of variance (ANOVA) followed by Tukey’s post‐hoc test (*Gapdh* mRNA *P* = 0.177 by one‐way ANOVA). Data in (B) were analysed using Welch’s ANOVA followed by Games‐Howell post‐hoc test (*Gapdh* mRNA *P* = 0.666 by one‐way ANOVA). *P ≤ 0.05 vs 0 min. #*P* ≤ 0.05, ##*P* ≤ 0.01 vs maximum levels. $*P* ≤ 0.05 vs minimum levels

Interestingly, many transcription factors exhibit an ultradian pattern of transcription in response to a pulse of ACTH.^21^ To determine whether CRTC isoforms also exhibited an ultradian pattern of transcriptional activation, hnRNA levels were measured in response to an ultradian pulse of ACTH (Figure 5A). Dynamic changes were detected in *Crtc1* (*F*_5,31_ = 3.574; *P* = 0.012) and *Crtc3* (*F*_5,31_ = 4.056; *P* = 0.006) hnRNA levels but not in *Crtc2* (*F*_5,31_ = 1.532; *P* = 0.209). We then investigated whether CRTC transcription will be affected by endotoxic stress (Figure 5B). hnRNA levels for all three isoforms were significantly altered following LPS injection (*Crtc1* *F*_8,13.64_ = 9.663; *P* = 0.004; *Crtc2* *F*_8,13.71_ = 2.679; *P* = 0.073; *Crtc3* *F*_8,13.82_ = 6.068; *P* = 0.003), peaking between 10 and 20 minutes and falling below basal by 60‐240 minutes.

## 4 DISCUSSION

In the present study, we demonstrate that nuclear translocation of endogenous CRTC2 and CRTC3 in the rat adrenal gland occurs in response to both basal, ultradian HPA axis activity, and following endotoxic stress, thus complimenting the findings from previous in vitro and in vivo studies in which activation of CRTC2 and CRCT3 was shown in response to ACTH and restraint stress.^13^, ^14^, ^16^, ^17^, ^18^ Here, we show that an increase in CRTC2 and CRTC3 nuclear localisation parallels, or precedes, the increase in CREB phosphorylation and *Star* transcription, as measured by *Star* hnRNA levels. Furthermore, using a ChIP assay, we confirm that association of CRTC2 and CRTC3 with the *Star* promoter occurs in vivo, and demonstrate clear stimulation of CRTC3 binding at the *Star* promoter in response to ultradian and endotoxic stress stimuli during periods of increased *Star* transcription. Furthermore, we show that levels of *Crtc1* and *Crtc3* transcription changes in response to ultradian HPA activity, whereas endotoxic stress leads to a prolonged down‐regulation of *Crtc1*, *Crtc2* and *Crtc3* transcription.

The observed changes in the levels of *Star* transcription are consistent with the results reported previously.^20^, ^21^, ^25^ *Star* hnRNA levels closely mimicked that of plasma CORT increasing within 5 minutes in response to a low dose ‘ultradian’ pulse and within 20 minutes following an endotoxic stressor. Similarly, peak nuclear localisation of endogenous CRTC2 and CRTC3 was seen at 5 minutes following an ultradian ACTH pulse, and this is consistent with the activation profile of CRTC2 following administration of a small dose of ACTH in methylprednisolone‐suppressed rats.^18^ We have previously demonstrated that activation of CRTC1, CRTC2 and CRTC3 occurs within 3‐7 minutes of ACTH treatment in adrenocortical cell lines,^16^ in contrast to earlier in vitro studies showing activation of CRTC2, although not of CRTC1 or CRTC3, in response to cAMP.^13^ Nuclear localisation of CRTC2 following HPA axis activation by restraint stress has previously been reported^17^ and, in the present study, we show that activation of CRTC2 and CRTC3 also occurs following endotoxic stress induced by LPS injection, with peak nuclear levels seen at 30‐60 minutes after exposure, returning to basal by 180 minutes.

Low dose ACTH and endotoxic stressor induced nuclear translocation of endogenous CRTC1, although with no statistical significance, suggesting that CRTC1 may still play a role in mediating ACTH‐induced *Star* transcription. This potentially indicates that CRTC1 is less responsive to ACTH signalling than CRTC2 or CRTC3. Elsewhere, CRTC1 has demonstrated a limited increase in nuclear translocation following forskolin treatment in hypothalamic 4B cells, HeLa cells and Hek293 cells.^15^, ^26^ Alternatively, constitutive nuclear localisation of CRTC1 may be responsible for the smaller increases in nuclear localisation detected following ACTH treatment. However, adrenal gland expression of CRTC1 is lower than CRTC2 or CRTC3 in the adrenal gland,^12^, ^16^ suggesting that the isoform is of limited importance in this tissue. Further functional studies are therefore required to establish the role CRTC1 plays in these cells.

Nuclear localisation of CRTC2 and CRTC3 tightly matches increases in *Star* hnRNA and CREB phosphorylation. This result supports a role for CRTC2 and CRTC3 in the initiation of *Star* transcription, through co‐activation of CREB. This relationship was further addressed by measuring the dynamics of CRTC2 and CRTC3 binding at the *Star* promoter. Although binding of CRTC2 at the *Star* promoter was detected by ChIP, no significant increase was detected following ACTH administration. This may suggest a potential for constitutive binding of CRTC2 under basal conditions. However, previous research investigating CRTC2 binding at the *Star* promoter in Y1 and ATC7‐L cells in vitro do not support this,^16^, ^27^ nor is there evidence for constitutive promoter binding of CRTC2 in other tissues.^28^, ^29^, ^30^ CRTC3 had increased binding by 5 minutes, when a peak in both nuclear CRTC3 and *Star* hnRNA levels was also observed. Thus, our data show that CRCT3 binding to the *Star* promoter occurs earlier than previously shown in vitro ^16^ (i.e. binding of CRTC2 and CRTC3 to the *Star* promoter by 30 and 15 minutes ACTH treatment, respectively). Similarly, binding of CRTC3 and a trend for binding of CRTC2 at the *Star* promoter showed peak levels at 30 minutes following an endotoxic stressor. Interestingly, although the amplitude of increases in pCREB levels were far greater following LPS administration compared to 10 ng of ACTH, the size of increase in both nuclear CRTC2 and CRTC3, as well as that of *Star* hnRNA levels, was of a similar magnitude following both an ultradian stimulus and an endotoxic stressor. This supports the hypothesis that initiation of ACTH‐induced *Star* transcription, is dependent on CRTC co‐activation of CREB, with phosphorylation of CREB alone being insufficient to potentiate *Star* transcription.^28^, ^31^, ^32^

With a similar pattern of activation seen in the present study for CRTC2 and CRTC3, our findings suggest that these isoforms play a similar role within ACTH‐mediated adrenal transcription, suggesting functional redundancy. We have previously shown that knockdown of either CRTC2 or CRTC3 in vitro resulted in similar levels of reduction of ACTH‐induced *Star* transcription, suggesting these isoforms act through a similar mechanism.^16^ Although expression of CRTC1 is largely confined to the central nervous system, CRTC2 and CRTC3 are expressed ubiquitously in peripheral tissues at comparative levels.^12^, ^33^, ^34^ Previous whole body knockout studies in mice have shown that knockout of both CRTC2 and CRTC3 causes embryonic lethality, with the presence of a single allele for either isoform required for viability.^35^ This emphasises the importance of these isoforms in mediating cAMP‐induced transcription and demonstrates that CRTC2 and CRTC3 play functionally redundant roles, at least in other key tissues. Further functional interrogation to determine the roles of these isoforms specifically in the adrenal is still required, potentially making further use of these animal knockouts.

Deactivation of CRTC2 and CRTC3 also occurred very rapidly following ACTH injection, and prior to decreases in nuclear pCREB and *Star* hnRNA, suggesting that termination of *Star* transcription is tightly linked to termination of CRTC and CREB activity. However, the delayed depletion of *Star* hnRNA levels may be the result of the processing time taken for hnRNA levels to be spliced into mature RNA, and this pattern of StAR hnRNA is similar to the 15‐minute delay between the detection of increased *Star* hnRNA and mRNA levels previously shown in Y1 cells.^13^, ^27^, ^36^ Similarly, following an endotoxic stressor, binding of CRTC2 and CRTC3 to the *Star* promoter declined after 30 minutes; however, CRTC3 binding was still significantly high at 2 hours and returned to basal by 3 hours, whereas *Star* hnRNA levels remain significantly high at 3 hours and returned to basal by 4 hours, suggesting that *Star* transcription continued via mechanisms involving other transcription factors and co‐regulators. Indeed, protein levels of SF‐1 have been demonstrated to increase by 6 hours after LPS injection,^37^ although not significantly by 4 hours.^21^ Levels of the dominant‐negative SF‐1 inhibitor, DAX‐1, however, were shown to be decreased between 3 and 4 hours, potentially leading to increased SF‐1 activity at this time.^21^ Furthermore, LPS has also been shown to rapidly increase rat adrenal hnRNA and mRNA levels of orphan nuclear receptors *Nr4a3* (aka Nor1) and *Nr4a1* (aka Nur77).^21^ Nur77 has been shown to bind the *Star* promoter by 1 to 4 hours of stimulation with cAMP in MA10 cells, although not at 30 minutes.^38^ Furthermore, Nor1 and Nur77 have been shown to up‐regulate *Star* promoter activity in MA10 cells,^39^ whereas both orphan receptors show similar patterns of activity in the adrenal gland.^40^ Studies into patterns of *Star* hnRNA splicing to mRNA in MA10 cells have also suggested a delayed secondary onset of transcription factors, such as Nur77 and C/EBPb, after 60 minutes of incubation with cAMP,^36^ with C/EBP comprising another known regulator of *Star* transcription,^41^, ^42^ up‐regulated by LPS in other tissues.^43^, ^44^, ^45^ The role of these factors and their interaction with the transcriptional complex during extended stress‐induced *Star* transcription will require further investigation.

Basal expression of CRTC isoforms appear to show little variation in the adrenal gland, with no significant changes in CRTC1, CRTC2 or CRTC3 protein levels across 24 hours. Similarly, following an ultradian ACTH pulse, *Crtc1‐3* hnRNA levels presented only small initial increases, with hnRNA levels falling significantly by 30 minutes. Conversely, other transcription factors, including SF‐1, Nur77 and Nor1, exhibit circadian variation in expression.^20^, ^21^ Furthermore, hnRNA levels of *Nr5a1*, *Nr4a1* and *Nr4a3* are all increased in response to 10 ng of ACTH, behaving similarly to *Star*.^21^ It appears that, as shown for CREB, CRTC activity is regulated by ACTH mainly at the level of post‐translational modifications. Interestingly, while no circadian variation in CRTC2 expression has been shown in the mouse SCN, a peak in *Crtc1* mRNA has been shown during the day.^46^ Following an endotoxic stressor, hnRNA levels for *Crtc1*, *Crtc2* and *Crtc3,* as well as levels of both cytosolic and nuclear CRTC2 and CRTC3 protein, decreased significantly below baseline, suggesting negative‐feedback on CRTC isoform expression in the adrenal gland as a result of the long‐term up‐regulation of *Star* transcription, Further studies are needed to explore this hypothesis.

In conclusion, our data show that CRTC2 and, more distinctly, CRTC3 appear to be key co‐transcription factors in the regulation of *Star* transcription, in response to both basal ultradian ACTH activity and following stress. Our results support a role for CRTC2 and CRTC3 to function alongside pCREB to mediate rapid adrenal *Star* transcriptional initiation, aiming to generate ultradian pulses of *Star* transcription and maintain basal StAR expression levels, as well as initiate *Star* transcription in response to HPA activation following endotoxic stress. However, these coactivators appear to play less of a role in the long‐term up‐regulation of the stress‐induced transcriptional response of the adrenal gland, indicating the participation of additional transcription factors. Although our findings have focused on measuring the expression of endogenous CRTC1‐3 and their patterns of activation in relation to HPA‐induced *Star* transcription, future research will require functional studies to allow the direct demonstration of this link.

## CONFLICT OF INTERESTS

The authors declare that they have no conflicts of interest.

## AUTHOR CONTRIBUTIONS

**Lorna Smith:** Formal analysis; Investigation; Methodology; Writing – original draft; Writing – review & editing. **Zidong Zhao:** Investigation. **Jamie Walker:** Formal analysis; Investigation. **Stafford Louis Lightman:** Conceptualisation; Funding acquisition; Supervision. **Francesca Spiga:** Conceptualisation; Formal analysis; Funding acquisition; Investigation; Methodology; Supervision; Writing – original draft; Writing – review & editing.

## Supporting information

Table S1‐S3

## Data Availability

The data that support the findings of this study are available from the corresponding author upon reasonable request.
