# Supplementary material for: Activation and expression of endogenous CREB‐regulated transcription coactivators (CRTC) 1, 2 and 3 in the rat adrenal gland
Source: J Neuroendocrinol. 2020 Dec 14;33(1):e12920. doi: 10.1111/jne.12920 (PMC7900988; doi:10.1111/jne.12920)
Supplement: Supplementary file 1 — Table S1‐S3 [file JNE-33-e12920-s001.pptx]

## Slide 1
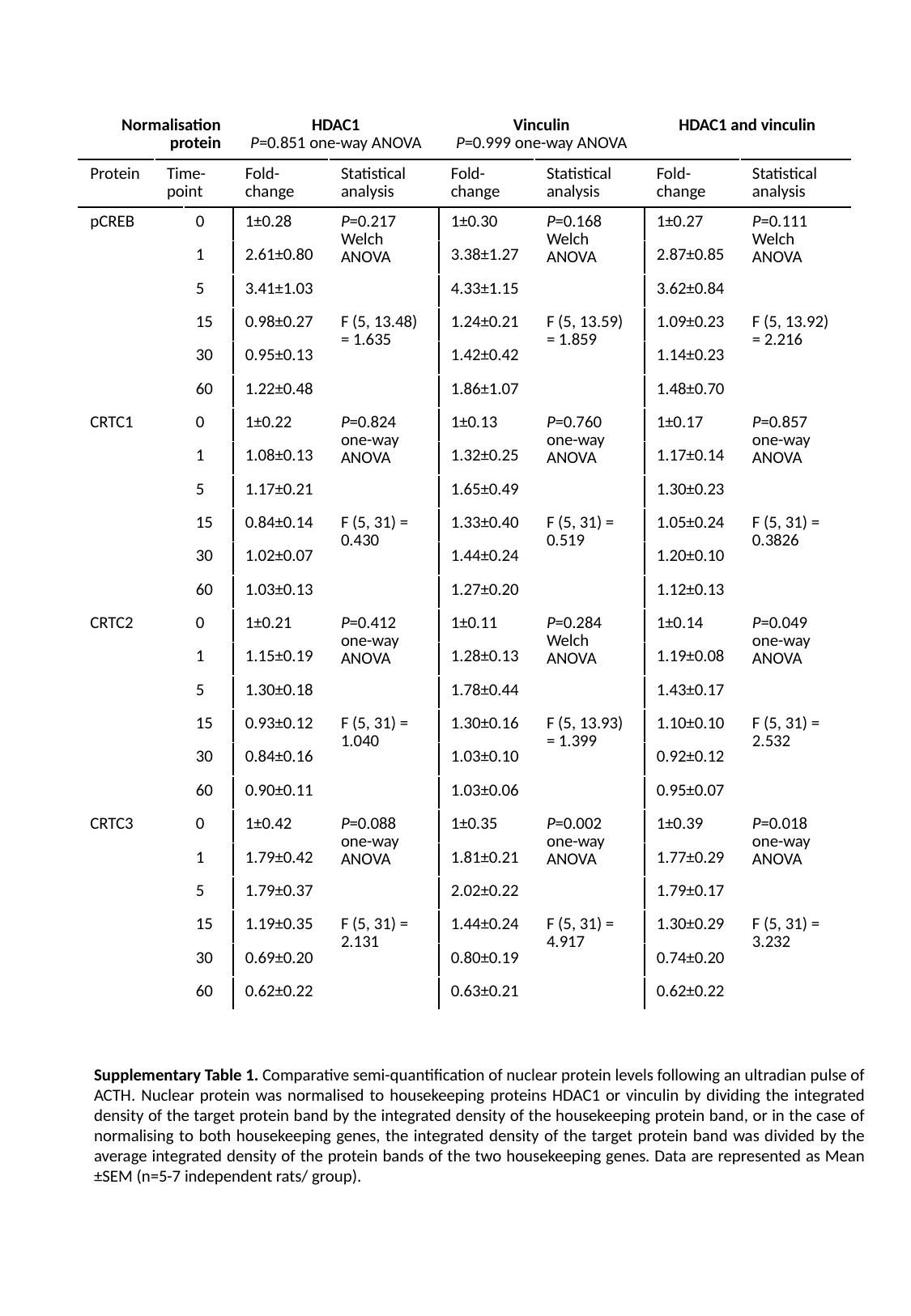

| Normalisation protein | | | HDAC1 P=0.851 one-way ANOVA | | Vinculin P=0.999 one-way ANOVA | | HDAC1 and vinculin | |
| --- | --- | --- | --- | --- | --- | --- | --- | --- |
| Protein | Time-point | Time-point | Fold-change | Statistical analysis | Fold-change | Statistical analysis | Fold-change | Statistical analysis |
| pCREB | | 0 | 1±0.28 | P=0.217 Welch ANOVA | 1±0.30 | P=0.168 Welch ANOVA | 1±0.27 | P=0.111 Welch ANOVA |
| | | 1 | 2.61±0.80 | | 3.38±1.27 | | 2.87±0.85 | |
| | | 5 | 3.41±1.03 | | 4.33±1.15 | | 3.62±0.84 | |
| | | 15 | 0.98±0.27 | F (5, 13.48) = 1.635 | 1.24±0.21 | F (5, 13.59) = 1.859 | 1.09±0.23 | F (5, 13.92) = 2.216 |
| | | 30 | 0.95±0.13 | | 1.42±0.42 | | 1.14±0.23 | |
| | | 60 | 1.22±0.48 | | 1.86±1.07 | | 1.48±0.70 | |
| CRTC1 | | 0 | 1±0.22 | P=0.824 one-way ANOVA | 1±0.13 | P=0.760 one-way ANOVA | 1±0.17 | P=0.857 one-way ANOVA |
| | | 1 | 1.08±0.13 | | 1.32±0.25 | | 1.17±0.14 | |
| | | 5 | 1.17±0.21 | | 1.65±0.49 | | 1.30±0.23 | |
| | | 15 | 0.84±0.14 | F (5, 31) = 0.430 | 1.33±0.40 | F (5, 31) = 0.519 | 1.05±0.24 | F (5, 31) = 0.3826 |
| | | 30 | 1.02±0.07 | | 1.44±0.24 | | 1.20±0.10 | |
| | | 60 | 1.03±0.13 | | 1.27±0.20 | | 1.12±0.13 | |
| CRTC2 | | 0 | 1±0.21 | P=0.412 one-way ANOVA | 1±0.11 | P=0.284 Welch ANOVA | 1±0.14 | P=0.049 one-way ANOVA |
| | | 1 | 1.15±0.19 | | 1.28±0.13 | | 1.19±0.08 | |
| | | 5 | 1.30±0.18 | | 1.78±0.44 | | 1.43±0.17 | |
| | | 15 | 0.93±0.12 | F (5, 31) = 1.040 | 1.30±0.16 | F (5, 13.93) = 1.399 | 1.10±0.10 | F (5, 31) = 2.532 |
| | | 30 | 0.84±0.16 | | 1.03±0.10 | | 0.92±0.12 | |
| | | 60 | 0.90±0.11 | | 1.03±0.06 | | 0.95±0.07 | |
| CRTC3 | | 0 | 1±0.42 | P=0.088 one-way ANOVA | 1±0.35 | P=0.002 one-way ANOVA | 1±0.39 | P=0.018 one-way ANOVA |
| | | 1 | 1.79±0.42 | | 1.81±0.21 | | 1.77±0.29 | |
| | | 5 | 1.79±0.37 | | 2.02±0.22 | | 1.79±0.17 | |
| | | 15 | 1.19±0.35 | F (5, 31) = 2.131 | 1.44±0.24 | F (5, 31) = 4.917 | 1.30±0.29 | F (5, 31) = 3.232 |
| | | 30 | 0.69±0.20 | | 0.80±0.19 | | 0.74±0.20 | |
| | | 60 | 0.62±0.22 | | 0.63±0.21 | | 0.62±0.22 | |
Supplementary Table 1. Comparative semi-quantification of nuclear protein levels following an ultradian pulse of ACTH. Nuclear protein was normalised to housekeeping proteins HDAC1 or vinculin by dividing the integrated density of the target protein band by the integrated density of the housekeeping protein band, or in the case of normalising to both housekeeping genes, the integrated density of the target protein band was divided by the average integrated density of the protein bands of the two housekeeping genes. Data are represented as Mean ±SEM (n=5-7 independent rats/ group).

## Slide 2
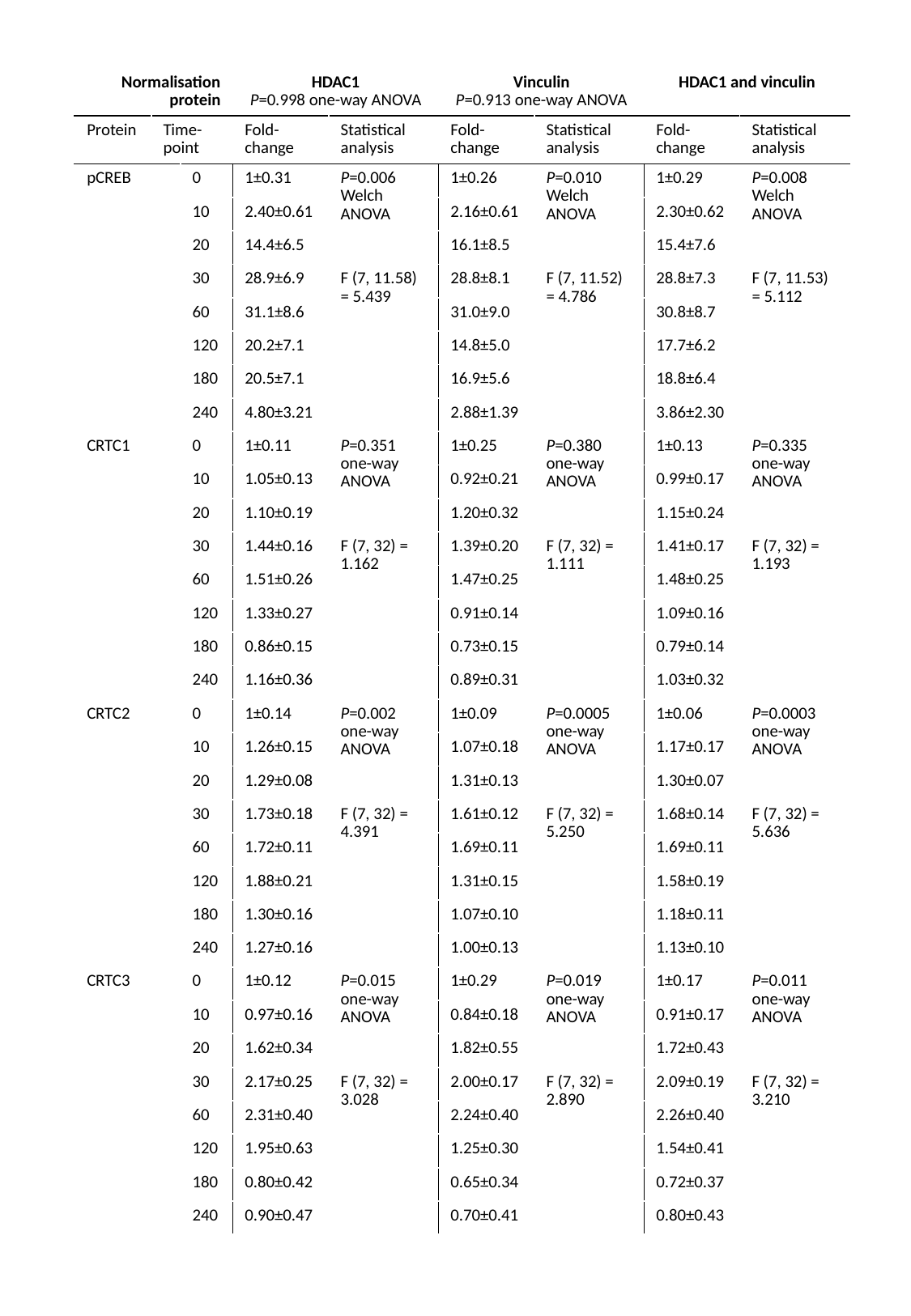

| Normalisation protein | | | HDAC1 P=0.998 one-way ANOVA | | Vinculin P=0.913 one-way ANOVA | | HDAC1 and vinculin | |
| --- | --- | --- | --- | --- | --- | --- | --- | --- |
| Protein | Time-point | Time-point | Fold-change | Statistical analysis | Fold-change | Statistical analysis | Fold-change | Statistical analysis |
| pCREB | | 0 | 1±0.31 | P=0.006 Welch ANOVA | 1±0.26 | P=0.010 Welch ANOVA | 1±0.29 | P=0.008 Welch ANOVA |
| | | 10 | 2.40±0.61 | | 2.16±0.61 | | 2.30±0.62 | |
| | | 20 | 14.4±6.5 | | 16.1±8.5 | | 15.4±7.6 | |
| | | 30 | 28.9±6.9 | F (7, 11.58) = 5.439 | 28.8±8.1 | F (7, 11.52) = 4.786 | 28.8±7.3 | F (7, 11.53) = 5.112 |
| | | 60 | 31.1±8.6 | | 31.0±9.0 | | 30.8±8.7 | |
| | | 120 | 20.2±7.1 | | 14.8±5.0 | | 17.7±6.2 | |
| | | 180 | 20.5±7.1 | | 16.9±5.6 | | 18.8±6.4 | |
| | | 240 | 4.80±3.21 | | 2.88±1.39 | | 3.86±2.30 | |
| CRTC1 | | 0 | 1±0.11 | P=0.351 one-way ANOVA | 1±0.25 | P=0.380 one-way ANOVA | 1±0.13 | P=0.335 one-way ANOVA |
| | | 10 | 1.05±0.13 | | 0.92±0.21 | | 0.99±0.17 | |
| | | 20 | 1.10±0.19 | | 1.20±0.32 | | 1.15±0.24 | |
| | | 30 | 1.44±0.16 | F (7, 32) = 1.162 | 1.39±0.20 | F (7, 32) = 1.111 | 1.41±0.17 | F (7, 32) = 1.193 |
| | | 60 | 1.51±0.26 | | 1.47±0.25 | | 1.48±0.25 | |
| | | 120 | 1.33±0.27 | | 0.91±0.14 | | 1.09±0.16 | |
| | | 180 | 0.86±0.15 | | 0.73±0.15 | | 0.79±0.14 | |
| | | 240 | 1.16±0.36 | | 0.89±0.31 | | 1.03±0.32 | |
| CRTC2 | | 0 | 1±0.14 | P=0.002 one-way ANOVA | 1±0.09 | P=0.0005 one-way ANOVA | 1±0.06 | P=0.0003 one-way ANOVA |
| | | 10 | 1.26±0.15 | | 1.07±0.18 | | 1.17±0.17 | |
| | | 20 | 1.29±0.08 | | 1.31±0.13 | | 1.30±0.07 | |
| | | 30 | 1.73±0.18 | F (7, 32) = 4.391 | 1.61±0.12 | F (7, 32) = 5.250 | 1.68±0.14 | F (7, 32) = 5.636 |
| | | 60 | 1.72±0.11 | | 1.69±0.11 | | 1.69±0.11 | |
| | | 120 | 1.88±0.21 | | 1.31±0.15 | | 1.58±0.19 | |
| | | 180 | 1.30±0.16 | | 1.07±0.10 | | 1.18±0.11 | |
| | | 240 | 1.27±0.16 | | 1.00±0.13 | | 1.13±0.10 | |
| CRTC3 | | 0 | 1±0.12 | P=0.015 one-way ANOVA | 1±0.29 | P=0.019 one-way ANOVA | 1±0.17 | P=0.011 one-way ANOVA |
| | | 10 | 0.97±0.16 | | 0.84±0.18 | | 0.91±0.17 | |
| | | 20 | 1.62±0.34 | | 1.82±0.55 | | 1.72±0.43 | |
| | | 30 | 2.17±0.25 | F (7, 32) = 3.028 | 2.00±0.17 | F (7, 32) = 2.890 | 2.09±0.19 | F (7, 32) = 3.210 |
| | | 60 | 2.31±0.40 | | 2.24±0.40 | | 2.26±0.40 | |
| | | 120 | 1.95±0.63 | | 1.25±0.30 | | 1.54±0.41 | |
| | | 180 | 0.80±0.42 | | 0.65±0.34 | | 0.72±0.37 | |
| | | 240 | 0.90±0.47 | | 0.70±0.41 | | 0.80±0.43 | |

## Slide 3
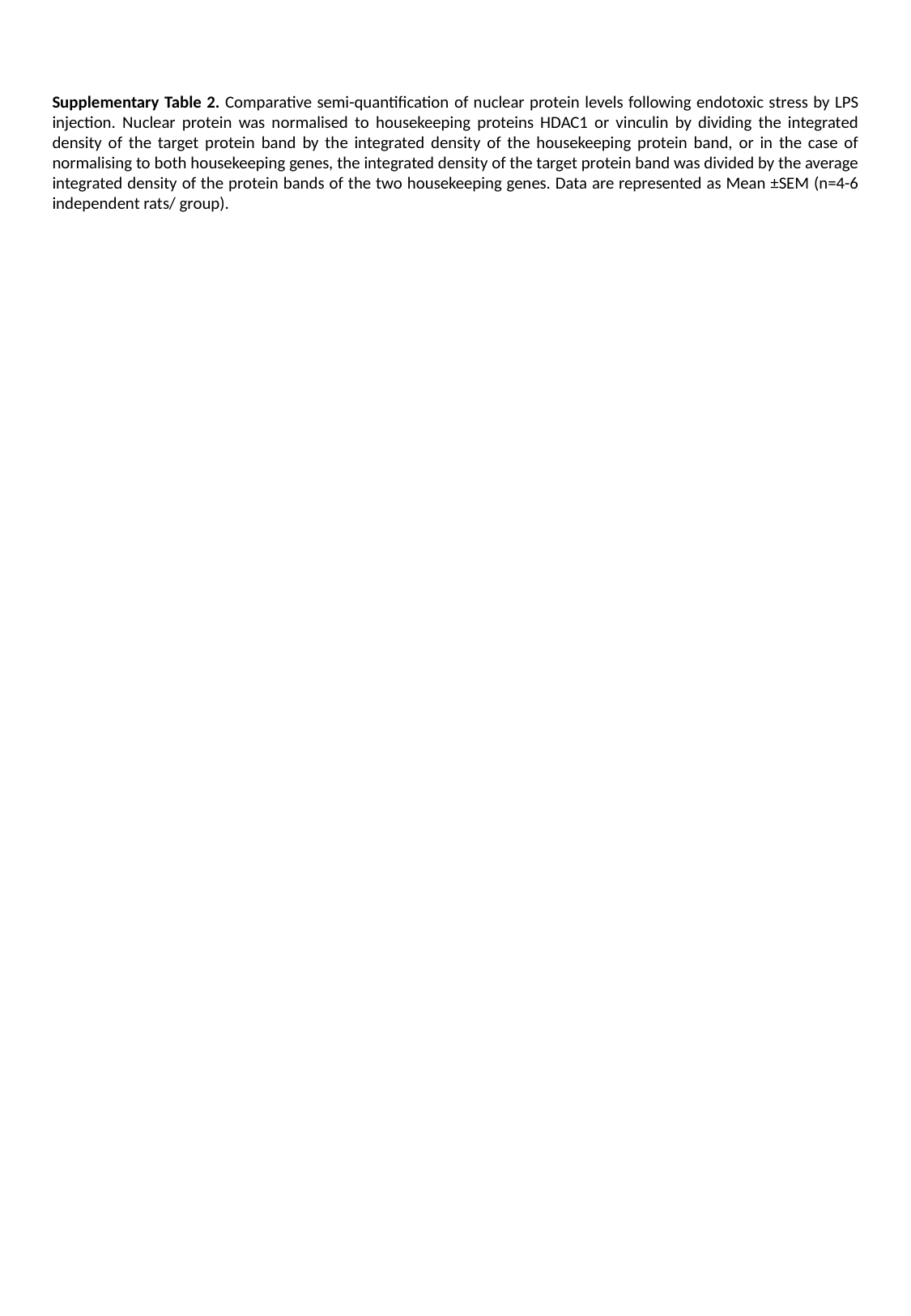

Supplementary Table 2. Comparative semi-quantification of nuclear protein levels following endotoxic stress by LPS injection. Nuclear protein was normalised to housekeeping proteins HDAC1 or vinculin by dividing the integrated density of the target protein band by the integrated density of the housekeeping protein band, or in the case of normalising to both housekeeping genes, the integrated density of the target protein band was divided by the average integrated density of the protein bands of the two housekeeping genes. Data are represented as Mean ±SEM (n=4-6 independent rats/ group).

## Slide 4
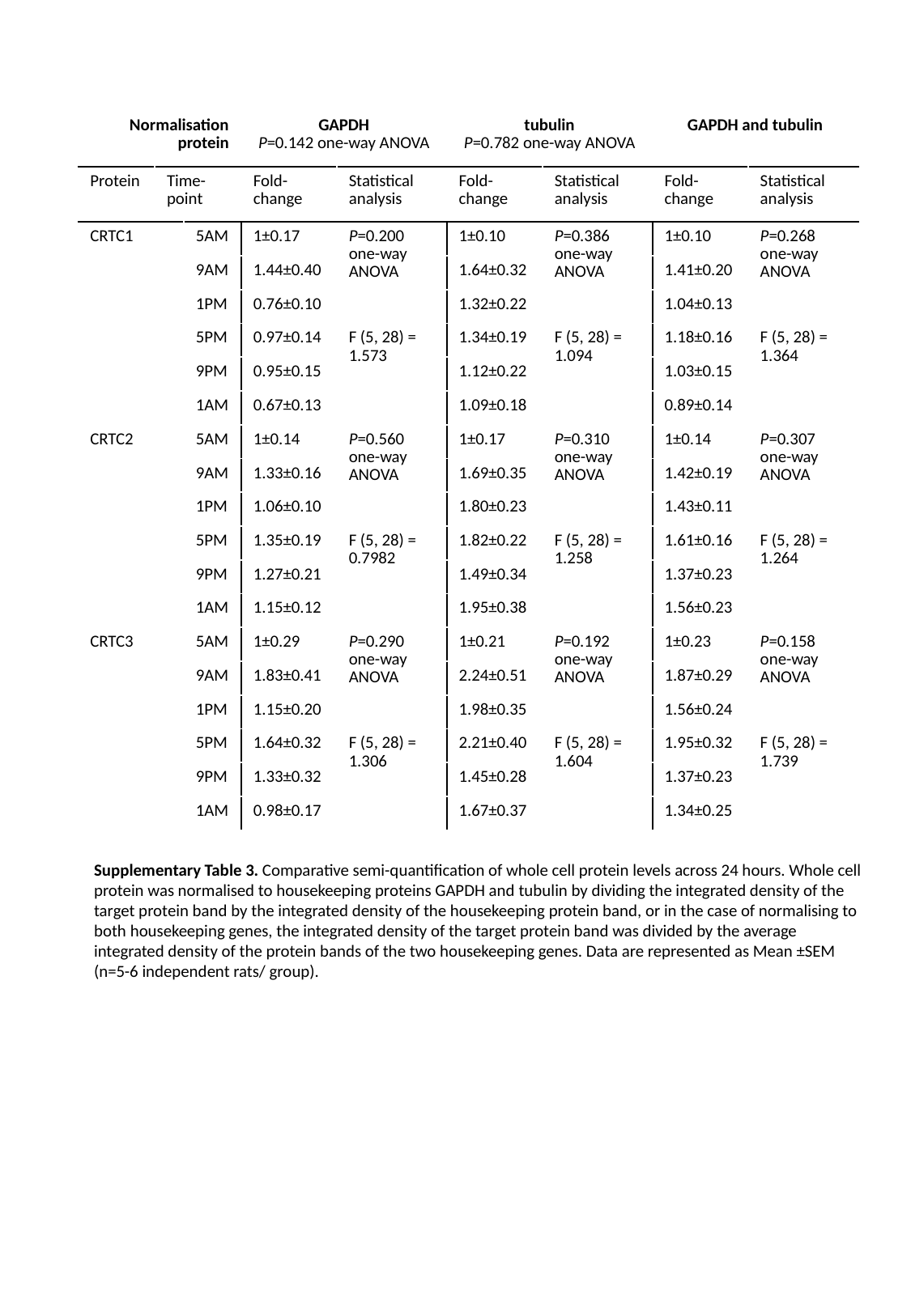

| Normalisation protein | | | GAPDH P=0.142 one-way ANOVA | | tubulin P=0.782 one-way ANOVA | | GAPDH and tubulin | |
| --- | --- | --- | --- | --- | --- | --- | --- | --- |
| Protein | Time-point | Time-point | Fold-change | Statistical analysis | Fold-change | Statistical analysis | Fold-change | Statistical analysis |
| CRTC1 | | 5AM | 1±0.17 | P=0.200 one-way ANOVA | 1±0.10 | P=0.386 one-way ANOVA | 1±0.10 | P=0.268 one-way ANOVA |
| | | 9AM | 1.44±0.40 | | 1.64±0.32 | | 1.41±0.20 | |
| | | 1PM | 0.76±0.10 | | 1.32±0.22 | | 1.04±0.13 | |
| | | 5PM | 0.97±0.14 | F (5, 28) = 1.573 | 1.34±0.19 | F (5, 28) = 1.094 | 1.18±0.16 | F (5, 28) = 1.364 |
| | | 9PM | 0.95±0.15 | | 1.12±0.22 | | 1.03±0.15 | |
| | | 1AM | 0.67±0.13 | | 1.09±0.18 | | 0.89±0.14 | |
| CRTC2 | | 5AM | 1±0.14 | P=0.560 one-way ANOVA | 1±0.17 | P=0.310 one-way ANOVA | 1±0.14 | P=0.307 one-way ANOVA |
| | | 9AM | 1.33±0.16 | | 1.69±0.35 | | 1.42±0.19 | |
| | | 1PM | 1.06±0.10 | | 1.80±0.23 | | 1.43±0.11 | |
| | | 5PM | 1.35±0.19 | F (5, 28) = 0.7982 | 1.82±0.22 | F (5, 28) = 1.258 | 1.61±0.16 | F (5, 28) = 1.264 |
| | | 9PM | 1.27±0.21 | | 1.49±0.34 | | 1.37±0.23 | |
| | | 1AM | 1.15±0.12 | | 1.95±0.38 | | 1.56±0.23 | |
| CRTC3 | | 5AM | 1±0.29 | P=0.290 one-way ANOVA | 1±0.21 | P=0.192 one-way ANOVA | 1±0.23 | P=0.158 one-way ANOVA |
| | | 9AM | 1.83±0.41 | | 2.24±0.51 | | 1.87±0.29 | |
| | | 1PM | 1.15±0.20 | | 1.98±0.35 | | 1.56±0.24 | |
| | | 5PM | 1.64±0.32 | F (5, 28) = 1.306 | 2.21±0.40 | F (5, 28) = 1.604 | 1.95±0.32 | F (5, 28) = 1.739 |
| | | 9PM | 1.33±0.32 | | 1.45±0.28 | | 1.37±0.23 | |
| | | 1AM | 0.98±0.17 | | 1.67±0.37 | | 1.34±0.25 | |
Supplementary Table 3. Comparative semi-quantification of whole cell protein levels across 24 hours. Whole cell protein was normalised to housekeeping proteins GAPDH and tubulin by dividing the integrated density of the target protein band by the integrated density of the housekeeping protein band, or in the case of normalising to both housekeeping genes, the integrated density of the target protein band was divided by the average integrated density of the protein bands of the two housekeeping genes. Data are represented as Mean ±SEM (n=5-6 independent rats/ group).
